# Aerosol therapy in relation to retinopathy of prematurity in mechanically ventilated preterm infants

**DOI:** 10.1186/s12890-019-0912-9

**Published:** 2019-08-13

**Authors:** Mei-Chin Yang, Hsiu-Feng Hsiao, Hsiu-Li Tseng, Ya-Wen Chiu, Yi-Hao Weng

**Affiliations:** 1 Department of Respiratory Therapy, Chang Gung Memorial Hospital, Chang Gung University, Taoyuan, Taiwan; 2 Master Program in Global Health and Development, College of Public Health, Taipei Medical University, Taipei, Taiwan; 3 Department of Pediatrics, Chang Gung Memorial Hospital, Chang Gung University College of Medicine, 199 Dunhua North Road, Taipei, 105 Taiwan

**Keywords:** GA, Aerosol therapy, ROP, HFV, iNO

## Abstract

**Background:**

Aerosol administration is increasingly being used as a therapeutic intervention for mechanically ventilated preterm infants. However, the effects of inhalation therapy on retinopathy of prematurity (ROP) have not yet been explored.

**Methods:**

A retrospective cohort study was conducted in a tertiary level neonatal intensive care unit (NICU) from 2011 to 2013. All preterm infants with a gestational age (GA) of 24~29 weeks receiving invasive intubation for more than 1 week in the NICU were included. Infants with severe congenital anomalies were excluded. ROP was defined as stage II or greater according to medical records by ophthalmologists. A multivariate logistic regression model was used to estimate the risk of ROP in relation to inhalation therapy after adjusting for confounders.

**Results:**

In total, 205 infants were enrolled in this study, including 154 with inhalation therapy and 51 without inhalation therapy. Univariate analyses showed an association of inhalation with the following characteristics: sex (*p* = 0.047), GA (*p* = 0.029), sepsis (*p* = 0.047), bronchopulmonary dysplasia (BPD) (*p* < 0.001), and ROP (*p* = 0.001). Furthermore, logistic regression analysis indicated that inhalation therapy was an independent risk factor for ROP (odds ratio (OR) = 2.639; 95% confidence interval (CI) = 1.050~6.615). In addition, infants with a GA of 24~25 weeks (OR = 6.063; 95% CI = 2.482~14.81) and 26~27 weeks (OR = 3.825; 95% CI = 1.694~8.638) were at higher risk of ROP than those with a GA of 28~29 weeks. Other factors – including sex, sepsis, BPD, and delivery mode – did not carry significant risk.

**Conclusion:**

Aerosol therapy with pure oxygen delivery is associated with ROP. Clinicians should exercise great caution when conducting aerosol therapy with excess oxygen in mechanically ventilated preterm infants.

## Introduction

Aerosol inhalation has been widely used to manage pulmonary diseases of children and adults. However, the distribution of particle deposition in the airway is different between infants and adults [1]. Recently, inhalation therapy is increasingly administrated to preterm infants. Several inhalants are used for mechanically ventilated infants, such as bronchodilators, corticosteroids, xanthine derivatives, mucoactive agents, and antibiotics. Inhaled bronchodilators help relieve airway constriction and improve lung compliance in premature infants with respiratory distress [2–4] and bronchopulmonary dysplasia (BPD) [5, 6]. The frequency and treatment duration of inhaled bronchodilators markedly vary because of no evidence-based guidelines [7]. In addition to bronchodilators, inhaled corticosteroids and xanthine derivatives have replaced systemic ones for managing BPD in preterm infants [8, 9]. Furthermore, the use of inhaled mucoactive medications makes secretions easier to transport and increases the efficiency of cough or mucus clearance, since secretion clearance is hampered by weakness and restrictive lungs [10]. However, the role of mucoactive agents in pulmonary critical care for mechanically ventilated preterm infants is not yet clear [11].

Retinopathy of prematurity (ROP) is a common disease of premature infants [12]. Exposing premature infants to supplemental oxygen is an important risk factor for developing ROP [13, 14]. Increased duration and number of hyperoxia events may increase the risk of ROP [15, 16]. Therefore, consensus guideline for resuscitation of preterm infants has been revised to deliver a low fraction of inspired oxygen [17].

Excess oxygen may be delivered to premature infants during inhalation therapy. However, any correlation of ROP with inhalation therapy has never been determined. Thus in the current study, we explored whether excess oxygen via inhalation therapy can cause ROP in extremely premature infants. The findings of this study provide clinical implications for therapeutic strategies to avoid the development of ROP.

## Materials and methods

### Enrollment of subjects

This retrospective study was conducted from June 1, 2011 to December 31, 2013 in the neonatal intensive care unit (NICU) of Chang Gung Memorial Hospital, a tertiary referral center in northern Taiwan. During this study period, preterm infants (with a gestational age (GA) of 24~29 weeks) who received invasive intubation for more than 7 days in the NICU of Chang Gung Memorial Hospital were included. The exclusion criteria included neonates with significant congenital malformation and referral neonates who had been admitted to another NICU for more than 1 day. Infants who did not receive screening for ROP were not enrolled, including death before ROP screening.

### Aerosol therapy

Infants who received aerosol administration of therapeutic drugs for at least 1 week during the first month of life were categorized as the aerosol therapy group. Inhaled medications were delivered through a commercially disposable nebulizer by a jet (100% compressed oxygen forced through a small hole into an adjacent reservoir containing medication in solution) [18]. When the aerosols were delivered to the endotracheal tube, the fraction of oxygen concentration was measured to estimate the final fraction of inspired oxygen (FiO_2_). The average readouts of FiO_2_ in the endotracheal tube were 48.1% when receiving 21% of oxygen, 52.0% when receiving 25% of oxygen, 56.9% when receiving 30% of oxygen, 58.7% when receiving 40% of oxygen, 62.4% when receiving 50% of oxygen, 68.0% when receiving 60% of oxygen, 77.7% when receiving 70% of oxygen, 84.1% when receiving 80% of oxygen, and 92.3% when receiving 90% of oxygen. Aerosol therapy was routinely performed for a period of 15~20 min every 8 h.

### Data collection

Clinical characteristics of enrolled subjects were recorded, including demographic characteristics, perinatal history, and outcomes. Mortality was defined as death after the screening of ROP at 1 month of age. Screening for ROP was performed by trained pediatric ophthalmologists using indirect ophthalmoscopy. The first time for ROP screening was 1 month of age. In this study, an infant with ROP was defined as stage II or greater for at least one eye by the International Classification of ROP [19].

### Statistical analyses

Statistics were compiled using a commercially available program (SPSS 19.0 for Windows, SPSS, Chicago, IL, USA). Categorical variables were analyzed using the Chi-squared test or Fisher’s exact test. For comparison of quantitative variables between groups, the null hypothesis that there was no difference between groups was tested by a one-way analysis of variance (ANOVA). A multivariate logistic regression model was used to estimate the risk of ROP in relation to inhalation therapy after adjusting for possible confounders. Odds ratio (OR) and 95% confidence interval (CI) were expressed after adjusting for the control variables. Significance was defined as *p* < 0.05.

## Results

**Fig. 1:**
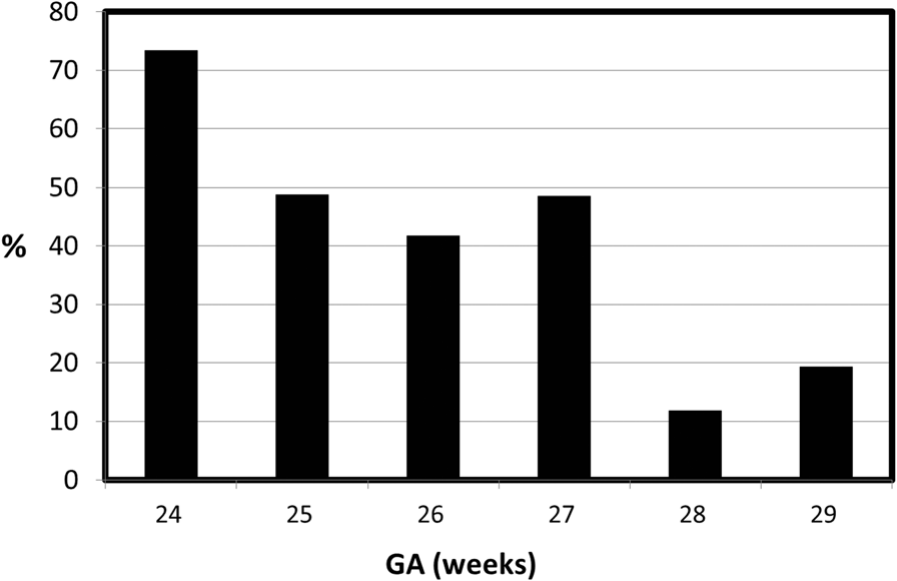
Incidence of ROP by GA (*N* = 205)

In total, 205 preterm infants were eligible for enrollment, including 54 infants with a GA of 24~25 weeks, 69 infants with a GA of 26~27 weeks, and 82 infants with a GA of 28~29 weeks. The incidence of ROP was 55.6% in infants with a GA of 24~25 weeks, 44.9% in those with a GA of 26~27 weeks, and 14.6% in those with a GA of 28~29 weeks (Fig. 1).

There were 154 infants (75.1%) who received aerosol therapy via an endotracheal tube. Drugs used for aerosol therapy included bronchodilators (ipratropium bromide, fenoterol, and salbutamol) and mucoactive agents (acetylcysteine and sodium bicarbonate). The most commonly used inhalation drug was acetylcysteine (99/154 = 64.3%). The use for bronchodilators and mucoactive agents was to decrease airway resistance and to promote mucin breakdown, respectively [20].

Demographic characteristics of enrolled infants are shown in Table 1. There were significant differences in sex and GA between infants with and without aerosol therapy. Males were more common among infants with aerosol therapy. Furthermore, the incidence of aerosol therapy was inversely proportional to GA: 85.2% at GA of 24~25 weeks, 78.2% at GA of 26~27 weeks, and 65.9% at GA of 28~29 weeks. Overall, the mortality rate after 1 month of age was 5.4%. There was no significant difference in the mortality between the aerosol and control groups. All deaths in both groups were related with sepsis.

**Table 1:** Demographic data of enrolled participants (*N* = 205)

Aerosol therapy	No	Yes	*p* value
Number	51	154	
Sex (%)			0.047
Male	22 (19.5)	91 (80.5)	
Female	29 (31.5)	63 (68.5)	
Delivery mode (%)			0.136
Vaginal	30 (31.3)	108 (68.7)	
Cesarean section	21 (21.7)	46 (78.3)	
GA (week) (%)			0.029
24~25	8 (14.8)	46 (85.2)	
26~27	15 (21.7)	54 (78.3)	
28~29	28 (34.1)	54 (65.9)	
Mortality	2 (3.9)	9 (5.8)	0.735

Table 2 illustrates the association between ROP and settings of mechanical ventilation. There was a significant correlation of ROP with the following factors – aerosol therapy, high frequency ventilation (HFV), inhaled nitric oxide (iNO), and the duration of intubation. In specific, ROP was more common in infants with aerosol therapy, iNO, use of HFV longer than 1 week, and intubation period more than 6 weeks. The relationship between ROP and aerosol therapy by three categories of GA is shown in Table 3. There was a trend of increasing ROP among infants with aerosol therapy in all categories of GA.

**Table 2:** Relationship between ROP and settings of mechanical ventilation (*N* = 205)

ROP	Yes	No	*p* value
	*N* = 73 *n* (%)	*N* = 132 *n* (%)	
Aerosol therapy			0.001
Yes	65 (89.0)	89 (67.4)	
No	8 (11.0)	43 (32.6)	
Duration of HFV (day)			< 0.001
Never use	15 (20.5)	65 (49.2)	
1 ~ 7	19 (26.1)	33 (25.1)	
8 ~ 28	15 (20.5)	14 (10.6)	
> 28	24 (32.9)	20 (15.1)	
iNO			0.009
Yes	10 (13.7)	5 (3.8)	
No	63 (86.3)	127 (96.2)	
Duration of intubation (day)			< 0.001
8 ~ 21	11 (15.1)	44 (33.5)	
22 ~ 42	20 (27.4)	53 (40.5)	
43 ~ 84	15 (20.5)	15 (11.5)	
> 84	27 (37.0)	19 (14.5)

**Table 3:** Relationship between ROP and aerosol therapy by GA (*N* = 205)

ROP	Yes	No	*p* value
Aerosol therapy (GA 24 ~ 25 wk., *N* = 54)	*N* = 30 (%)	*N* = 24 (%)	0.120
Yes	28 (93.9)	18 (75.0)	
No	2 (6.7)	6 (25.0)	
Aerosol therapy (GA 26 ~ 27 wk., *N* = 69)	*N* = 31 (%)	*N* = 38 (%)	0.385
Yes	26 (83.9)	28 (73.7)	
No	5 (16.1)	10 (26.3)	
Aerosol therapy (GA 28 ~ 29 wk., *N* = 82)	*N* = 12 (%)	*N* = 70 (%)	0.051
Yes	11 (91.7)	43 (61.4)	
No	1 (8.3)	27 (38.6)	

A risk assessment of ROP was conducted using a multivariate logistic regression model (Table 4). Adjusted confounders included sex, aerosol therapy, GA, iNO, HFV, and intubation period. The regression analysis showed that inhalation therapy carried a greater risk of ROP. In addition, infants with a GA of 24~27 weeks were at greater risk of ROP than those with a GA of 28~29 weeks. Other factors – including sex, HFV, iNO, and intubation period – did not carry significant risk.

**Table 4:** Risk assessment for ROP by a logistic regression analysis

Risk factor	OR	95% CI	*p* value
Male	0.592	0.301~1.165	0.129
Aerosol therapy	2.828	1.069~7.484	0.036
iNO	2.084	0.585~7.426	0.257
GA (week)			
24~25	4.001	1.518~10.55	0.005
26~27	3.920	1.688~9.104	0.001
28~29	reference		
Duration of HFV (day)			
> 28	1.610	0.566~4.580	0.372
8 ~ 28	2.111	0.719~6.197	0.174
1 ~ 7	1.823	0.763~4.357	0.177
Never use	reference		
Duration of intubation (day)			
> 84	1.971	0.569~6.832	0.285
43 ~ 84	1.224	0.342~4.373	0.756
22 ~ 42	0.838	0.311~2.259	0.726
8 ~ 21	reference		

## Discussion

This study depicts the association between aerosol therapy and ROP among mechanically ventilated preterm infants. To our best knowledge, this is the first study to investigate the potential impact of aerosol therapy on the risk of ROP. Our findings suggest that aerosol administration of therapeutic drugs via an endotracheal tube in combination with pure oxygen may result in harmful effects of ROP in infants with a GA of 24~29 weeks.

ROP is a multifactorial disease. One of the most significant risk factors for ROP is a low GA, as shown in our and other studies [12]. Immature vascularization induces an increased susceptibility of the retina to oxidative damage. Therefore, our study enrolled infants with a GA of 24~29 weeks. Furthermore, our study aimed to clarify the possible impact of aerosol therapy on ROP. Thus, we only investigated infants who received aerosol therapy before the development of ROP.

In this study, inhalants were delivered with 100% oxygen via a nebulizer. It is well documented that high oxygen saturation can increase the risk of ROP [21]. However, a lower target range of oxygenation may result in an increase in mortality [22]. Therefore, adequate oxygen during aerosol therapy is mandatory to maximize benefits and minimize harms. To reduce the potential risk of ROP, several factors should be considered while conducting aerosol therapy. Our data suggest that an oxygen blender or metered dose inhaler-spacer device may be needed for inhalation therapy to reduce excess oxygen exposure [23]. In addition, using the mechanical ventilator or a mesh nebulizer to produce the aerosol can decrease the fraction of oxygen concentration. Furthermore, the total time outside the target saturation range is associated with the development of ROP [16]. Thus, shortening the duration or frequency of high-oxygen inhalation may be helpful in decreasing the risk of ROP.

The role of oxygen in the development of ROP is complex. Many reports showed a reduction in ROP with supplemental oxygen in a later course of treatment after ROP had already developed [24, 25]. Accordingly, aerosol therapy with pure oxygen may be fine when an infant already has ROP.

In this study, we did not focus on the medications used in aerosol therapy. One would question whether medicines such as acetylcysteine may have an impact on the development of ROP. Our study found no significant difference in ROP between inhalants with and without acetylcysteine (data not shown). Furthermore, it has been shown that acetylcysteine may have a protective effect against ROP [26]. Thus we believe that ROP is not simply modulated by inhaled medicines, although the potential effects of inhalation drugs cannot be completely ruled out.

In our study, the univariate analyses showed ROP was more common in infants with HFV, iNO, and prolonged intubation. However, the multivariate model did not find a significant correlation of ROP with these ventilation settings, which are in accordance with previous reports of meta-analysis illustrating that HFV and iNO did not increase the risk of ROP in preterm infants [27, 28].

There are limitations to our study. First, this study was a retrospective design. Randomized controlled trials are needed to determine the causal effect of aerosol therapy on ROP. Second, the effectiveness of aerosol therapy for premature neonates was not evidence-based [21]. No clear long-term benefits have been demonstrated yet. Third, we did not investigate the potential impact of delivery devices. Nevertheless, the delivery devices were not changed throughout the study period.

## Conclusion

Aerosol therapy is increasingly administered to premature infants during the first hospital month. Possible adverse effects of aerosol therapy need to be elucidated. Our study implies an increased risk of ROP with aerosol medicines administrated using pure oxygen. Appropriate oxygen delivery may play a role in reducing the progression of ROP. Clinicians should use great caution when conducting aerosol therapy with excess oxygen in preterm infants.

## Data Availability

Please contact Yi-Hao Weng for data requests.

## Abbreviations

BPD: Bronchopulmonary dysplasia
GA: Gestational age
HFV: High frequency ventilation
iNO: Inhaled nitric oxide
NICU: Neonatal intensive care unit
ROP: Retinopathy of prematurity
